# Effectiveness of Non-Pharmacological Methods, Such as Breastfeeding, to Mitigate Pain in NICU Infants

**DOI:** 10.3390/children9101568

**Published:** 2022-10-17

**Authors:** Zoi Koukou, Anatoli Theodoridou, Eleftheria Taousani, Angeliki Antonakou, Eleftherios Panteris, Styliani-Stella Papadopoulou, Anna Skordou, Stavros Sifakis

**Affiliations:** 1 School of Health Sciences, International Hellenic University (IHU), 57400 Sindos Thessaloniki, Greece; 2 Laboratory of Forensic Medicine and Toxicology, School of Medicine, Aristotle University of Thessaloniki, 54124 Thessaloniki, Greece; 3 Department of Obstetrics and Gynecology, Mitera Hospital, 71202 Heraklion, Greece

**Keywords:** NICU, breast feeding, pain management, non-nutritive sucking, oral sucrose, pain scales

## Abstract

Neonates do experience pain and its management is necessary in order to prevent long-term, as well as, short-term effects. The most common source of pain in the neonatal intensive care unit (NICU) is caused by medically invasive procedures. NICU patients have to endure trauma, medical adhesive related skin injuries, heel lance, venipuncture and intramuscular injection as well as nasogastric catheterization besides surgery. A cornerstone in pain assessment is the use of scales such as COMFORT, PIPP-R, NIPS and N-PASS. This narrative review provides an up to date account of neonate pain management used in NICUs worldwide focusing on non-pharmacological methods. Non-steroidal anti-inflammatory drugs have well established adverse side effects and opioids are addictive thus pharmacological methods should be avoided if possible at least for mild pain management. Non-pharmacological interventions, particularly breastfeeding and non-nutritive sucking as primary strategies for pain management in neonates are useful strategies to consider. The best non-pharmacological methods are breastfeeding followed by non-nutritive sucking coupled with sucrose sucking. Regrettably most parents used only physical methods and should be trained and involved for best results. Further research in NICU is essential as the developmental knowledge changes and neonate physiology is further uncovered together with its connection to pain.

## 1. Introduction

Historically, until the 19th century, the dominant view was that newborns could not perceive pain. The American Medical Association in 1848 states that newborns are unable to remember a painful stimulus [1]. Health professionals in the 80′s used to believe that newborns did not feel pain as children and adults do [2]. Instead, they thought that newborns’ responses to discomfort were muscle reflexes [2]. Furthermore thought out the 20th century, an effort to avoid anesthetic and analgesic drug side effects, resulted in newborns being exposed to pain. Instead, anesthesiologists were administering oxygen in combination with nitric acid and muscle relaxants [3]. The management of neonatal pain was revolutionized by technical studies in the early 1980s, where the above pain reduction technique, also known as the Liverpool technique, was essentially disproved, as the research showed that the addition of an analgesic to anesthesia resulted in stress reduction due to reduced pain [4]. In today’s era there are in place pain management and assessment protocols, as research has proven that there are significant neurodevelopmental consequences of under-treating pain in neonates [5]. International guidelines have now addressed neonatal pain [6,7,8,9,10].

### Nutrition and Neurodevelopmental Issues

Contrarily to past practices and beliefs, newborns can indeed feel and understand pain stimulus, with premature infants been even more susceptible to pain [11]. Heavy pain for the newborn can affect physiologically all the main organ systems with potential severe consequences in later on [11]. Neonatal nervous systems are in continuous development, thus, they are prone to neurodevelopmental changes from painful stimuli [12], with effects evident even in adult life [13,14]. Even more so for premature neonates that are put into neonatal intensive care units (NICU) in order to become fully grown infants. Their requirements for stimuli and nutrients are at the foetus level and need to be highly regulated to reach the appropriate level of development [15]. NICU has seen many advance in the past years but, accommodation in one does not secure the required development especially for very low birth weight (<1500 g) infants [16,17]. Stunted growth is directly linked to neurodevelopmental problems [18,19] that in turn are linked to worse sensory management for preterm neonates, making them more sensitive to pain [20].

Nutritional practices in NICU are increasingly incorporate breast feeding in their regimes considering it a medical intervention for the correct development of the neonate [21,22,23,24]. Human milk is not the only milk supplied nor breast feeding the only delivery method. Cow’s milk and enriched milk products are also utilized but human milk has many health inductive properties that make it more that nutritional and indeed more medicinal in nature [25,26,27,28]. Breastfeeding releases antioxidant and anti-inflammatory substances to the infant [29], helps establish the gut microbiome and the immune system [30,31] while promoting neurological development [32]. An additional and equally important reason is the required parental presence enhancing the parental-infant interaction that is also an indicator for a succesful outcome after NICU [28].

This narrative review provides an up to date account of neonate pain management used in NICUs world-wide focusing on non-pharmacological methods and the effectiveness of breastfeeding in particular. Academical databases such as PubMed and Google Scholar were queried for the latest information in neonatal pain management studies and reviews focusing on NICU and non pharmaceutical methods.

## 2. Pain and Fetal Life

Pain in the fetus is transmitted by mechanisms unique and different from those in adults. In the 8th week of pregnancy the connections of the periphery with the spinal cord are formed, while in the 10th week the C fibers are formed in the spinal cord [33]. A waiting zone under the cortex then develops at week 15, which is necessary to carry out important sensory functions of the fetus [34]. During the 17th week, the development of the cerebral cortex takes place, which continues even after birth. In addition, data obtained from studies on the behavior of the fetus, prove that most of the time the fetus “sleeps” inside the uterus, however during painful stimuli fetuses were stimulated [35]. It is important to note that fetuses show stress-like hormonal responses to pain during intrauterine surgeries performed at 16 weeks of gestation, suggesting that a pain perception pathway has been formed at that time [35]. Therefore, it becomes important to limit painful procedures for fetuses in order to prevent a potential impact of pain and stress both on survival and on its long-term neurodevelopmental course [36].

### 2.1. Types of Pain

Newborns can undergo over 300 painful procedures while at hospital. [11]. Blood test, vaccinations, vitamin injections, heel sticks, minor surgery such as circumcision, are painful and invasive procedures that are done daily [12]. These procedures are performed in a much higher frequency in NICU for preterm or sick term infants [20,37,38].

An adverse neurobiological effect has been found in preterm infants who have been exposed to stress and pain [39]. The stay of newborns and especially premature ones in the neonatal intensive care unit results in them receiving frequent painful processes which are classified as acute or chronic, the impact of which is pain with different pathophysiology [12]. These two types of pain have been associated with cognitive delays, neurodevelopmental effects and more specifically with reduction of the head, brain function and finally its sensory areas. Chronic pain and stimulation in neonatal life affects the developing brain [39]. In addition, there are also some direct effects such as oxidative stress, increased heart rate and low weight gain in the premature 32 weeks of pregnancy [40].

#### 2.1.1. Neonatal Trauma of NICU (IMTN)

Many times, the environment of the unit and more specifically the high noise, the intense lighting and the numerous procedures of the medical and nursing staff can put the newborns at risk affecting the emotional, behavioral and cognitive results [41,42]. Early life initiation of both preterm and term infants in the NICU may affect their neurodevelopmental and psychological outcomes due to exposure to prolonged stressors such as parental separation. Neurodevelopmental effects may take months to years to become apparent [41]. However, newborns with greater prematurity become more vulnerable and depending on the degree of illness they suffer from. An important impact on the life of the newborn is stress, and especially chronic stress that can cause problems in the structure of the brain [41]. Magnetic resonance imaging helped in the discovery that stress, especially in premature babies, was responsible for brain immaturity [20]. In addition, parental separation is considered the most important stressful factor with three categories of intensity (1) short ≤15’ (2) prolonged ≤3 h (3) in deprivation ≤ 24 h [43]. The term Infant Medical Trauma in the NICU (IMTN) [44] is even used to describe this potentially traumatic experience in NICU that can lead to neurodevelopmental effects. Autism is 3 times higher in former premature infants [45]. Furthermore, prematurity combined with IMTN has been shown to be responsible for psychological disorders and elevated rates of depression and anxiety throughout childhood and into adulthood [46].

When the trauma is caused in the early stages of life and the newborn does not have a stable caregiver the possibility of its developmental trajectory being affected is increased [47]. Thankfully, not all newborns perceive trauma to the same extent, evidence shows that they can have some resistance to it [48].

#### 2.1.2. Skin Injuries Associated with MARSIs

The daily medical and nursing interventions in NICU, without the use of the appropriate techniques for the placement and removal of adhesive gausses, can cause skin trauma, termed MARSIs (medical adhesive related skin injuries), which affects the quality of life of the newborns, as its consequence is the causing pain at the site of damage [49]. The skin of newborns and especially premature newborns becomes extremely sensitive. Premature babies have thinner skin especially, the outer layer [50], with fewer cellular connections and reduced epidermal cellularity, an alkaline PH and a lower concentration of the natural moisturizing factor (NMF) [51]. Skin injuries can involve irritations, contact or allergic dermatitis, folliculitis, skin infections and other complications. Aggravating factors that play an important role in increased susceptibility to skin injury are considered: malnutrition and dehydration, skin drying, overlong moisture exposure, certain medications, phototherapy and repeated use of adhesive products [49]. The use of adhesives in the form of self-adhesive gausses, was the leading cause of skin breakdown among NICU neonates. Pediatric patients have similar incidences with MARSIs attributed to adhesive tape used to place endotracheal intubation [52].

The goal of healthcare professionals should be to prevent injury. However, in order to achieve this, it is important, according to the latest data, to use fewer irritating products, such as hydrogel pads, silicone-based or hydrocolloid pads [53] or even new technologies for pain monitoring [54]. At the same time, the systematic evaluation of their application points and their frequent change of position should be ensured In addition, we avoid great pressure on the patch points by changing positions of the newborn in the incubator and we follow the protocols for correct fixation of the equipment (e.g., nasogastric catheter) [55,56].

## 3. Pain Scales

However, pain prevention is important for hormonal, behavioral and physiological outcomes [11]. Since pain can be initiated anywhere, from the spinal cord, the skin, the organs or induced by a pathological condition such as meningitis, necrotizing enterocolitis and osteomyelitis, its need to be identified and evaluated [57]. The evaluation in neonates is evidently non-communicational, thus, is carried out using pain scales that follow specific pain indicators, behavioral, such as sound pitch, facial expressions, physiological like heart rate, blood pressure, oxygen levels etc., or developmental like gestational age or a combination of them [58]. Nevertheless, physiological indicators are considered most appropriate to assess pain in distressed neonates or neonates with neurological problems. The most reliable indicator is heart rate, followed by oxygen saturation and increased intracranial pressure [13].

In terms of behavioral indicators, facial expressions are the most common, such as frowning, hermetically closing the eyes, the depth of the nasolabial fold and the open mouth. As an indicator it shows stability of presence at all ages of newborns and is considered the “gold standard ” for pain assessment [13]. According to a study carried out by Slater et al., the above indicators seem to be associated with cortical activation after a painful stimulus [59]. As for the biochemical and hormonal indicators, it was found after taking a saliva or plasma samples, that newborns undergoing surgical operations and painful procedures have an increased concentration of cortisol [13]. Other individual factors that affect the evaluation indices in newborns are, gestational age of the newborn [60], previous and prolonged exposure to and number of previous painful procedures [13].

Up to now, more than 40 pain rating scales for neonates in NICU have been published [58,61]. They are a mainstay as they help us not only quantify pain, but can provide an accurate depiction of the impact of pharmacological and non-pharmacological pain management interventions in a neonate. Based on a recent systematic review on neonatal pain performed in 2021, there are still no fully objective pain assessment tools to assess pain/stress in NICU [58]. In a very detailed systematic review with a focus on NICU randomized trials, of 29,137 newborns from different countries, about 20 assessment scales were reviewed [58]. The most used scales were: Premature Infant Pain Profile (PIPP)/PIPP—Revised [62,63] in 43.9% of the trials; Neonatal Infant Pain Scale (NIPS) [64] in 23.9%; Neonatal Facial Coding System (NFCS) [65] in 9.4%; Acute pain rating scale (APN, DAN in French) [66] 5.7% and only 4.3 for the well-known COMFORTneo (including both COMFORT and COMFORT-B) [67,68], finally Neonatal Pain, Agitation, and Sedation Scale (N-PASS) [69], was the last of the most commonly used with 2.8%. Table 1 has more information about those commonly used scales in NICU.

The vast majority of studies focused on procedural pain only, with the most common pain scales being PIPP or PIPP—Revised [63], and NIPS [64]. The results showed that the most common pain was procedural, with heel lance, venipuncture and intramuscular injection being the most common painful procedures [58]. There were an equal number of studies investigating pharmacological and non-pharmacological interventions. The most frequently reported interventions were sucrose or glucose administration, followed by local anesthetics and morphine [58].

Another systematic review in newborns and infants this time by Giordano et al., focused on validation of pain and sedation scales [70]. They found that only 28 of the 65 scales had been statistically tested for validity, consistency, and interrater reliability. Concluding, they stressed the clinical need for precise and verified threshold valuesforpain for any scale [70].

To sum up, pain in newborns in the unit must be evaluated completely objectively. Therefore, the use of validated pain scales is the best method available, based on the individualized application to each newborn. No scale is considered the “gold standard” with universal application in all cases of pain [58]. For example, while NIPS is only validated for acute pain is very much preferred. for ongoing and postoperative pain as well [58]. Additionally, it is worth noting that most studies do not report much data on postoperative pain and ongoing pain/stress, as the researchers’ attention is focused on interactive pain [58]. Most of the indicators used in the scales are subjective in nature, a fact that makes staff training particularly important [58].

The review of the protocols and thorough training of the NICU staff becomes necessary at regular intervals, as new studies bring to light modifications of the scales according to the newest data, having better clinical results and less subsequent effects on the health of the newborn [58].

## 4. Non-Pharmacological Methods

Non-pharmacological methods, such as non-nutritive sucking [71], skin-to-skin care [72,73], swaddling/facilitated tucking [74], rocking/holding [73,75], and music [76,77] have been recently found to be effective as pain relief strategies in infants in the NICU. In addition, Shah, et al. (2012) found administering glucose/sucrose offered similar pain relief to breastfeeding in neonates [76].

Table 2 summarizes all the common pain management methods in NICU.

### 4.1. Non-Nutrient Sucking

Non-nutritive sucking (NNS), is the provision of a pacifier or the sucking of the fingers or the hand in neonates of NICU. NNS is considered a safe and effective method of pain relief during the pinprick procedure in neonates [71]. But it is more effective in conjunction with sucrose/glucose sucking [94]. A randomized controlled clinical trial with cross-over design [97] in an Iranian NICU with 60 infants demonstrated the effectiveness of using oral dextrose for pain management during a heel prick in comparison to facilitated tucking. They did find also that facilitated tucking is effective compared to no management and can be utilized in constrained situations or in combination with oral dextrose [97]. According to a recent study [98], where sucrose was compared with non-nutritive suction, it appeared that with the help of the NIPS scale, sucrose was superior in reducing the duration of crying when removing adhesive patches from newborn wounds. But it was noted that sucrose alone is not superior to behavioral pain management compared to the combination of methods [98]. A significant reduction in pain scores was found in neonates with NNS as well as sucrose administration compared to NNS or sucrose alone in a 2022 study [94] albeit for mild pain caused. Short duration of the sucrose administration pain relief effect has been reported previously [95,96]. NNS usage in the contex of Point of care quality improvement method (POCQI) using a commercially fixed dosage oral sucrose solution gave a 96% rise in NNS use, in a level 3 NICU in India [99].

### 4.2. Breastfeeding

Breastfeeding in NICU has to be initiated and then established for the neonates to automate the process by tube and then progress to oral feeding after they are developed enough [21,100,101]. When established bottle-feeding and breastfeeding are the most common delivery methods for maternal milk even though exclusive breastfeeding is the gold standard recommended for at least first six months [102,103].

Breastfeeding utilization in the NICU becomes highly important, as studies are published daily with the properties and benefits of breast milk [104,105]. Newborns undoubtedly need their mother’s contact and proper nutrition for their future development. Breastfeeding for a duration of more than 2 min prior to a painful procedure [85] is a valid non pharmacological pain management method. While, the presence of the mother to breastfeed, especially in very premature infants is not applicable, it is a valid option in more grown infants [85].

Direct-breastfeeding is the direct suckling on the breast regardless of the delivery of milk to the infant [106], while expressed breast feeding is the extraction and storage of milk for later delivery with a bottle [107]. Direct breastfeeding is the unequivocal best practice in non-pharmacological pain management methods since it has been compared to all other methods and has been found more effective [61]. It fared much better compared to swaddling [76,86], maternal holding [87] or skin-to-skin care [88,89], topical anesthetics [90] and cooling sprays [91], non-nutritive sucking [92] and music [93] in pain management.

A well-known Cochrane systematic review and meta-analysis [76] from 20 studies (1075 direct and 996 expressed breast feeding infants) established the pain management effectiveness of breastfeeding either direct or in full-term infants. A later systematic review [108] for 15 studies with 1908 infants in total, was more explicit in their results.

Direct breast-feeding was stated as the best method of non-pharmacological pain management compared to all other (holding, skin-to-skin contact, topical anesthetics, and music), and was preferable even to administration of glucose/sucrose in full-term infants [76]. While they did not recommend expressed breast milk as they deemed it not effective enough for pain relief [76].

### 4.3. Non-Pharmacological Methods Used by Parents

Despite the effectiveness of non-pharmacological methods for procedural pain management in infants, being evident [109], we know precious little about the actual methods the NICU infant parents use. Campbell-Yeo et al. (2011) believed that such strategies are mostly used by nurses to hold on to NICU authority over infant caregiving, despite parents wishing to be more engaged in comforting their infants [85]. Parental involvement in infant pain management in NICU has been previously addressed and needs to be higher [110,111].

A unique cross-sectional and descriptive study of 178 parents whose newborns were placed in NICUs in Finland [112] found that most parents almost exclusively used physical methods, such as touching, holding, and positioning. Very few used other established NICU strategies such as breastfeeding, with only 2% of the parents utilizing it and NNS with oral sucrose (6%). They stated that parental pain management was relate to newborn condition and gestational age [112]. Parents did not use many valid strategies, such as swaddling, facilitated tucking/kangaroo care, music, breastfeeding, and NNS/sucrose [112]. Parents used easy to copy and perform methods that did not require to be taught by the nursing staff, clearly lacking training and knowledge on these effective, yet more difficult to master, strategies [112]. They concluded with a plea to extend parents’ use and knowledge of non-pharmacological pain management methods to manage their infants’ procedural pain in the NICU [112].

The majority of non-pharmacological pain management methods are more effective performed by the parents rather than NICU staff [85]. Thus, NICU staff and healthcare professionals must enable parents to follow such methods, by providing guidance and training for a more active involvement into their child’s care while in NICU [113,114].

## 5. Pharmacological Methods

Historically, several accepted pain management methods over the years have changed due to undesirable clinical results [6]. It is generally accepted that the use of pharmacological methods in NICU is a controversial issue, as the goal of the medical and nursing staff is not only to deal with short term pain, but also to properly manage the incident, in order to mitigate the subsequent consequences in the long term [115]. In addition, it is worth noting that most non-steroidal anti-inflammatory drugs (NSAIDs) are not recommended for infants < 6 months of age, due to established adverse side effects [80]. The most widely used NSAID is paracetamol [77] for mild to moderate pain relief, and to reduces the need to use morphine [116], thus reducing the risk of opioid addiction. Intubation and mechanical ventilation are usually the procedures that opioids such as fentanyl and morphine are used for, since they are causes for persistent pain [6]. Recent research is inconclusive whether opioids have an effect on pain and neurodevelopmental outcomes at later age [117,118]. Also morphine or fentanyl usage probably has limited effectiveness on reducing the duration of mechanical ventilation and neonatal mortality [119].

Midazolam, and its family of substances benzodiazepines are in use in NICU especially for sedation. As they are found to strengthen opioid effectiveness in causing respiratory depression and hypotension safety concern have been raised [81]. Several other substances some controlled, methadone [78], ketamine, propofol, and dexmedetomidine, where considered, but very limited data and known side effects have restricted their use [79].

**Figure 1:**
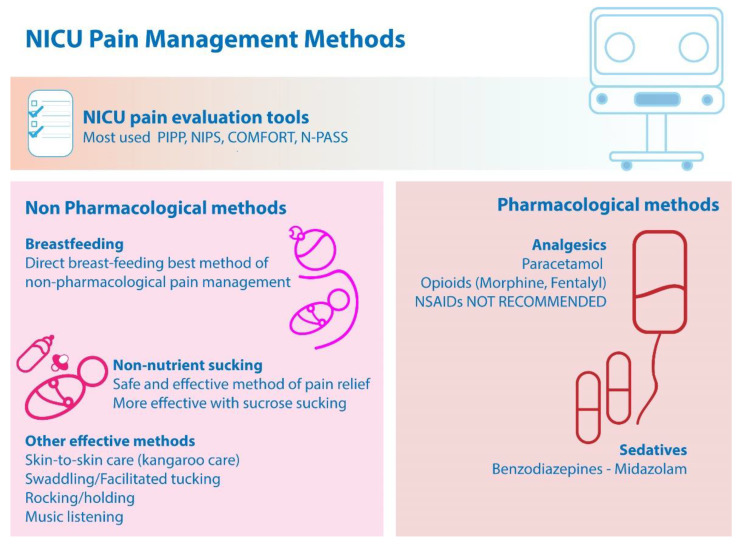
Main findings on NICU pain management methods.

In a recent study it was shown that the use of morphine allowed enhanced pain relief compared to its combination with midazolam in NICU, with a lower cost. Thus, morphine alone stands as a common analgesia strategy especially in neonates with respiratory distress syndrome (RDS) [120]. Figure 1 summarizes the main findings.

## 6. Discussion

The assessment and management of neonatal pain by healthcare professionals is a very important step to optimally prevent short-term and long-term consequences. Historically, the prevailing opinion was that newborns do not feel pain, however, they perceive pain and react to the painful stimulus albeit differently than older children [1,11]. However fetuses are now believed to be able to feel pain even as early as the eighth week when connections of the periphery to the spinal cord are formed [120].

In order to assess the type of pain intensity and other parameters, the use of approved scales, based on the updated guidelines, is essential to allow for an objective evaluation. While there is no gold standard when it come to the scales [58] we recommend keeping abreast of the current research and only use updated versions of any chosen scales but with rigorous training in their use [58]. In addition to evaluation, pain management is equally important. In addition to pharmacological methods [6], the neonatal population can also accept non-pharmacological ones, which must be a primary goal to avoid negative side effects [6]. The most widely used substance is paracetamol, [77], while depending on the severity of the situation, opioids, local anesthetics, fast-acting anesthetics and inhaled sedatives are also well utilized in NICU.

Due to the negative pharmacological effects, the use of non-pharmacological methods is preferable [115]. Non pharmacological methods, most notably, breastfeeding [85], non-nutritive sucking [71], swaddling, skin-to-skin care [72], facilitated tucking, rocking/holding [73,75], and music [82] are effective ways to manage pain in NICU neonates.

Non-nutritive and or sucrose/glucose suckling are well studied and established methods that are helpful [98] albeit for mild pain [94]. Breastfeeding especially direct, has been found to be the best practice in non-pharmacological pain management [61], compared all other methods [76,86,87,88,89,90,91,92,93]. A tiered approach to pain management is advisable dictated by the procedure to be performed [121].

Most of non-pharmacological pain management methods are more effective performed by the parents rather than NICU staff [85] in appropriate conditions. Regrettably most parents used only physical methods, such as touching, holding, and positioning [112]. The established NICU strategies discussed here are not know to parents or are not suggested to them [112], There is a clear need to extend parents’ use and knowledge of non-pharmacological pain management methods to manage their infants’ procedural pain in the NICU [112] as further indicated by a recent systematic review on the clinical practive guidelines [10] by providing guidance and training for a more active involvement into their child’s care while in NICU [113,114] and in using pain scales and adequate methods of pain prevention or relief depending on pain severity. Also POCQI methodology for the acceptance of new methods in NICU as illustrated [99] can be an equally powerful tool for pain management by training and conditioning NICU staff on short but precise processes, that can be passed on to parents as well.

## 7. Conclusions

It’s of the outmost importance to point out the fact that the cornerstone in the management of neonatal pain is the timely provision of pharmacological and non-pharmacological methods with respect to the individual needs and condition of the NICU patient. The primary goal in each case is the application of non-pharmacological methods with the fewest medical interventions to alleviate additional adverse effects. Strict indication or avoidance of painful procedures and on bundling procedures (if possible) in order to reduce the number of painful events should always be advocated as well as a tiered approach to pain management. However, in case of failure of adequate analgesia, pharmacological methods are used, always knowing the subsequent effects on the health of the newborn. Thus, the best pain management method that is also the best nutritional option as well, is maternal breastfeeding when possible, followed by non-nutritive and sucrose/glucose suckling.

Further study on the management of pain in NICU is essential as the developmental knowledge changes and neonate physiology is further uncovered together with its connection to pain. 

## Figures and Tables

**Table 1:** Most used Pain Scales in Infants and the NICU.

Scale	Use	Indicators Used	Scoring
PIPP [62]	Premature infants in NICU	3 behavioral (facial actions: brow bulge, eye squeeze, nasolabial furrow), 2 physiological (heart rate and oxygen saturation), and 2 contextual (GA and BS) items	Seven-item, four-point scale 21 points for preterm infants <28 weeks GA and 18 points for full-term infants
PIPP-R [63]	Extremely low gestational age (ELGA) infants	Same as PIPP	Seven-item, four-point scale
NIPS [64]	Infants < 1 year old	5 behavioral, (facial expression, cry, arms, legs, and state of arousal) 1 physiological factor (breathing patterns),	Score 0–7, Score > 3 is indicative of pain
NFCS [65]	premature neonates, term-born neonates, and infants ≤18 months of age	10 behavioral, (brow bulge, eye squeeze, nasolabial furrow, open lips, horizontal mouth stretch, vertical mouth stretch, taut tongue, lip purse, chin quiver, tongue protrusion.) Top 3 (brow bulge, eye squeeze, and nasolabial furrow) suffice for pain assessment.	Score 0–10 for premature infants: 10 Score 0–9 full term infants
COMFORTneo [67,68]	Premature infants in NICU	6 behavioral, (alertness, calmness, muscle tone, physical movement, facial tension, and respiratory behavior/crying). Respiratory behavior in ventilated patients and Crying in nonventilated patients.	6 items are scored on a 5-point scale, ranging from 1 to 5, with total score ranging from 6 to 30.
N-PASS [69]	NICU ventilated and/or postoperative infants 0–100 days of age, ≥ 23 weeks of gestation	3 behavioral, (behavior/state, facial expression, extremities/tone) 4 physiological vital signs (heart rate, respiratory rate, blood pressure and/or oxygen saturation)	Score 0–10

GA: Gestational age, BS: behavioral state, NICU: neonatal intensive care unit, PIPP: Premature Infant Pain Profile, PIPP-R: Premature Infant Pain Profile-Revised, NIPS: Neonatal Infant Pain Scale, NFCS: Neonatal Facial Coding System, N-PASS Neonatal Pain, Agitation, and Sedation Scale.

**Table 2:** Most common pain management methods in NICU.

Pharmacological Methods	Type of Pain Management
**Analgesics**	
Paracetamol [77]	Mild to moderate pain
Opioids, mostly Morphine & Fentanyl [6]	Persistent pain
Methadone [78], ketamine, propofol, dexmedetomidine [79].	Persistent pain, limited use
Non-Steroidal Anti-Inflammatory Drugs [80]	Not recommended for infants < 6 months of age, due to established adverse side effects
**Sedatives**	
Benzodiazepines- Midazolam, [81]	Sedation
**Non-pharmacological methods**	**Type of Pain Management**
Non-nutritive sucking [71]. Provision of a pacifier or the sucking of the fingers or the hand during painful event	Acute procedural Mild to moderate pain
Skin-to-skin care (kangaroo care) [72,73]. Newborns wearing only a diaper being held next to their mother’s bare chest	Acute procedural Mild to moderate pain
Swaddling/Facilitated tucking [74] Wraping the infant tightly/Holding the infant in the side-lying, flexed fetal-type position by hand	Acute procedural Mild pain
Rocking/holding [73,75]. Holding the neonate and swaying in an rocking motion	Acute procedural Mild pain
Music listening [82], Recorded maternal singing [83] White noise/classical music playing during painfull procedures [84]	Pasification, Recovery reinforcement of sucking, Acute procedural pain and stress relief
Breastfeeding for a duration of more than 2 min prior to a painful procedure [61,76,85,86,87,88,89,90,91,92,93]	Acute procedural pain mild to medium
Oral administration of Sucrose/glucose [94,95,96]	Acute procedural pain mild to medium, short lived duration

## Data Availability

Not applicable.
